# Assessing the Prevalence of SARS-CoV-2 in Free-Living and Captive Animals

**DOI:** 10.3390/pathogens11121405

**Published:** 2022-11-23

**Authors:** Hinh Ly

**Affiliations:** Department of Veterinary and Biomedical Sciences, College of Veterinary Medicine, University of Minnesota, Twin Cities, MN 55108, USA; hly@umn.edu; Tel.: +1-612-625-3358

Several animal species, including cats, dogs, hamsters, mink, big cats, great apes and white-tailed deer, etc., have been shown to be susceptible to natural SARS-CoV-2 infections (for a review, see [1]). The concern is whether they can act as reservoirs through which the virus can evolve before being transmitted to humans. Indeed, a previous study has shown that SARS-CoV-2 can circulate and genetically evolve in farmed mink, making the viral spike protein less readily susceptible to neutralization by human SARS-CoV-2 antibodies [2]. Inter- and intraspecies transmissions of such a virus in mink farms have been documented [3]. Animal caretakers on a mink farm in Denmark were found to contract SARS-CoV-2, which subsequently spread to the local human population, based on the similarity in the viral genomic sequences analyzed. When the virus has an opportunity to evolve in a reservoir host, spillback events of the virus into human populations can pose a significant risk to public health [4,5]. 

Recent studies have shown that free-ranging animal species, such as white-tailed deer, are highly susceptible to SARS-CoV-2 infection and transmission (i.e., intraspecies transmission). For example, 35.8% (129/360) of nasal swabs performed on white-tailed deer in Ohio (USA) were found to be positive for SARS-CoV-2 as detected by RT-PCR (reverse transcription polymerase chain reaction) [6]. The authors of this study were also able to detect probable intraspecies transmission among deer that were infected with the SARS-CoV-2 variants found to be prevalent among the residents of Ohio at the time of testing. However, there was no evidence of spillback of the virus into the human population in that case. Supportive evidence for intraspecies transmission was strengthened in a study showing that young deer (fawns) could be infected by SARS-CoV-2 under the laboratory setting and that they could shed viruses and infect other fawns in adjacent pens [7]. Another study showed that free-ranging white-tailed deer in 24 of the 30 US states tested (as well as in some Canadian provinces bordering the US) harbored SARS-CoV-2 [8]. Whether other wildlife species are also susceptible to SARS-CoV-2 infections remains unknown. 

In a recent report published in *Pathogens* [9] and selected as an editor’s choice article in 2021, the authors used blood samples, muscle extracts and/or feces of wild boars (*Sus scrofa*), red foxes (*Vulpes vulpes*) and European jackals (*Canis aureus moreoticus*) collected from June to the end of December 2020 in Croatia to test for evidence of SARS-CoV-2 infections. Additionally, the investigators used blood and cloacal swabs collected from yellow-legged gulls (*Larus michahellis*) in Croatia for SARS-CoV-2 testing. In total, 153 blood and 93 fecal samples from wild boars, 204 muscle extracts and 94 fecal samples from red foxes, 65 muscle extracts and 33 fecal samples from jackals, and 111 blood and cloacal swabs from yellow-legged gulls were tested. The investigators used a commercially available enzyme-linked immunosorbent assay (ELISA) kit for the detection of anti-N (anti-nucleocapsid) antibodies of SARS-CoV-2. They then used a commercially available virus neutralization test (sVNT) to assess the anti-SARS-CoV-2-neutralizing antibodies (NAbs) in the blood and muscle extracts of the animals that were found to be positive by the ELISA. To detect SARS-CoV-2 RNA, the investigators used real-time RT–PCR.

Serological results showed that six wild boars (3.9%), six red foxes (2.9%) and three jackals (4.6%) tested positive for anti-SARS-CoV-2 antibodies in the ELISA, but that all these N-based ELISA-positive samples tested negative for NAbs in the sVNT assay. However, four red fox samples appeared to show weakly positive results initially but were negative in subsequent tests. Based on various laboratory criteria, the authors concluded that those that initially tested positive were probably false-positive results. This was corroborated by the fact that RNA samples from wild boars, red foxes, jackals, and yellow-legged gulls all tested negative for SARS-CoV-2. Based on these results, the authors concluded that no spillovers of SARS-CoV-2 occurred in these wild animal species during the second wave of COVID-19 in the human population in Croatia. The difference in results produced by the N-based ELISA used in this study might partly be due to the nature of the commercial test kit used in the analysis, as a previous study has shown that the quality of the recombinant N protein used to develop the ELISA is critical [10]. It is unclear how the recombinant N protein was expressed and purified for use in many of the commercial kits, including the one used in this study [9].

It is interesting to note that the investigators of this study [9] also tested fecal samples of various zoo animals, including armadillos, monkeys (capuchin, howler), gibbons, chimpanzees, lemurs, Egyptian fruit bats, big cats (lynxes, African lions, leopards), wolves, mongooses, meerkats, European bears, otters, red pandas, dwarf pigs, peccaries, hippopotami, camels, alpacas, llamas, goats and sheep, and found no evidence of SARS-CoV-2 infection in any of these animals, even in those (mammals) that were in contact with known SARS-CoV-2 RNA-positive handlers. This result is unique, as other reports indicated that zoo animals that were in contact with SARS-CoV-2-infected handlers, including large cats and great apes, were found to be susceptible. A tiger in the Bronx Zoo in New York (USA) was shown to contract SARS-CoV-2 from a zoo employee who tested positive for SARS-CoV-2 [11]. Subsequently, several other tigers and lions in the same zoo were also found to be positive. Similarly, other big cats (African lions, Amus tigers and a Sumatran tiger) tested positive for the virus, presumably from their interactions with a SARS-CoV-2-positive zookeeper [12]. At the San Diego Zoo Wildlife Alliance (USA), a troop of western lowland gorillas were also positively diagnosed with SARS-CoV-2, possibly due to their frequent contact with SARS-CoV-2-positive handlers [13]. As a result, some captive animals in different zoos in the USA have been vaccinated against SARS-CoV-2 infection [14,15]. However, additional efforts to screen certain wild and domestic animals for SARS-CoV-2 are warranted as there is now ample evidence demonstrating that many of them are susceptible to natural SARS-CoV-2 infections and transmissions.
